# Monograph I. On the Cerebral Diseases of Children, with Regard to Their Early Manifestations and Treatment

**Published:** 1848-10-01

**Authors:** Walter C. Dendy

**Affiliations:** LATE PRESIDENT OF THE MEDICAL SOCIETY OF LONDON, SURGEON TO THE ROYAL INFIRMARY FOR CHILDREN, ETC.


MONOGRAPH I.

ON THE

### CEREBRAL DISEASES

### OF CHILDREN,

WITH

REGARD TO THEIR EARLY MANIFESTATIONS AND TREATMENT.

BY

WALTER C. DENDY, ESQ.

LATE PRESIDENT OF THE MEDICAL SOCIETY OF LONDON,

SURGEON TO THE ROYAL INFIRMARY FOR CHILDREN,

ETC.

LONDON:

JOHN CHURCHILL, PRINCES STREET, SOHO.

1848.

ON THE

### CEREBRAL DISEASES OF CHILDREN.

---

The study of infantile pathology is replete with interest. The excessive fatality which marks the spring-time of human life must constantly claim our most anxious exertions, while the simplicity which characterizes the diseases of childhood, uncomplicated by those disordered actions and changes so constant in after life, and uninfluenced by mental emotion, renders their study deeply interesting.

With this simplicity, however, there is combined predisposition and proneness to disease, as well as susceptibility of external impression in an eminent degree. The pink hue and extreme vascularity of the skin indicate an excess of arterial sensibility; while the irregular, almost convulsive action on very slight stimulation, the facility with which laughter and crying are induced, and the rapidity with which extreme exhaustion will often occur, are proofs of the intense sensibility with which the nervous system is endowed.

The disorders of children are also marked by extreme celerity of progress, illustrated by the rapidity with which inflammatory disease will often run its course, and the sudden formation of its morbid products, the highly elaborated lymph membrane of croup, and the almost instantaneous effusion of serum in the wasserschlag.

The mucous membranes are the earliest sufferers from extraneous excitement. The membrane of the alimentary canal has suddenly imposed on it the most important duty of assimilation; that of the respiratory canals is directly submitted to the influence of air of a low temperature. Notwithstanding, therefore, the profusion of mucus with which nature has supplied these tissues, these influences are the constant source of various disorders, especially diarrhœa, bronchitis, and pneumonia.

Happily, however, the mucous membranes are endowed with a faculty of relieving themselves by pouring out an excess of this natural secretion; they may therefore be often hailed as the *vis medicatrix.*

But it is the pathology of the infantile encephalon which is the subject of our most especial interest; so replete with sympathies, so delicate, sensitive, and impressible, so insidious and so obscure in its indications, notwithstanding all which Paisley, Whytt, Quin, Goëlis, Cheyne, Breschet, and others, have written. It is the seat, also, of that mind which is to influence the thoughts, passions, and actions of after life, while thus combined with matter; until at its appointed season it will

> "Burst a seraph in the blaze of day."

Thus will the condition of the infantile brain often mould the intellect of the adult, illustrating physically the poet's thought,

> "The child is father of the man."

Soon after the fœtal existence is changed, and the double circulation established, the organ of intellect commences its progressive state of development and growth. In the early period of man's life the brain seems composed almost of medullary matter, or tubular neurine; the grey matter, or hemispherical ganglion, exists but in the germ, and becomes pink, dusky, and reddish grey, in a direct ratio, probably, with the manifestations of intellect. I have seen, however, even in infancy, the grey matter in excess; too rarely, however, to induce conclusions. The brain is, indeed, the centre of our system. Not only is it the focus of the senses, the organ of intellect, but it is very early called on to assume the regulation of the organic functions; its sympathies, therefore, are universal, and its transition states must hence be incessantly subject to disturbing influences, and often replete with peril. Even its physiological changes must frequently be interrupted, and may illustrate much of its pathology. Thus, about the second year of childhood the natural diminution of tenuity and of aqueous particles in the brain, and the deposition of phosphorus, may perhaps account for the very early forms of serous effusion.

It is at the period especially of teething, and of the progress of cranial ossification, that the vascularity of the head is increased, to afford an adequate supply for those processes, and hence the frequent establishment of cutaneous eruptions on the head and face during the period of infancy and childhood. Dentition (the fatality of which is probably about six per cent.) is especially influential in lighting up, directly or indirectly, inflammatory action in the cerebral tissues, and developing those latent tendencies to disease which constitute *diathesis*. It is to these idiosyncrasies that I would most especially refer, as they constitute the great *predisposing causes* to cerebral disease. For although disorder may be lighted up in a previously healthy brain, yet it is the combination with these peculiar diatheses—as that of struma, for instance—which imparts so formidable, and often irremediable, a character to cerebral maladies.

To investigate, then, the nature of these predisposing causes, we must refer, not to the breathing child, but to the intra-uterine, or fœtal state, indeed, to the very moment of conception.

From that instant to the moment of birth, the peculiar states of the mother exert on the fœtus a most potent influence. Hereditary or constitutional taint, systemic debility or defect, thought, passion, emotion, and I may add, compression of the abdomen by the dress, may all tend to the disturbance of the perfect development of the ovum, whether this consist in evolution or epigenesis. Hereditary tendency has been often proved. Goëlis alludes to six abortions of dead hydrocephalic fœtuses at the sixth month, from a mother who also had three other children born alive, who subsequently became hydrocephalic. And Dr. Copland, in his Dictionary, alludes to other parallel instances. Without fully discussing the question physiologically, I could cite from my own memory many instances of this maternal influence, especially when sudden and deep mental impression has been made by any unfortunate or melancholy catastrophe—a truth easy to be believed, when we recollect the extreme interference of unhappy incidents with the common and more palpable functions of the body. If mental impression so affect gastric and intestinal function, why not that of the formative process? I was engaged to attend a lady residing at Kennington, the mother of two very healthy children. She progressed in her pregnancy most favourably, and in perfect health, until the seventh month, when her husband, from a sudden attack of hæmoptysis, dropped dead. She suffered a sort of rigor when the woful truth was imparted to her, and complained of excess of action in her child, and was sensible of a chilling sensation in the region of the uterus. From that moment I was suspicious of the future condition of the fœtus. It was at eight months and a week very quickly expelled. It is now (at seven years old) a confirmed idiot—a cretin; the height is that of a child of two years and a half old—partly occasioned by an excurvation of the dorsal vertebræ, so that its head, though placed on a *very* thick short neck, lops on its shoulders. Some of the cylindrical bones are curved, the epiphyses enlarged. It has only ten teeth, very small, and discoloured. It takes very little notice, but its eyes, instinctively as it were, follow a candle, even round and round when whirled before them, and at this time, and this only, there is a very faint expression of gratification.

Dr. Goëlis expresses his belief in the following sentences:—" Multiplied experience of the influence of terror and anxiety in the mother during the latter period of pregnancy, was afforded me and the other physicians of Vienna, in the year 1809, when our imperial city was bombarded. Most of the children who were born after this frightful catastrophe, in about ten, twenty, or thirty days after their birth, were seized with convulsions and died. Within the cranium were found traces of inflammation, and in the ventricles of the brain effusions of lymph and serum."

The truth of this influence is questionless, yet some pathologists still doubt and attempt to disprove this maternal impression—in a very natural opposition, perhaps, to the assertion of *special* influence—that fright or accident may produce in the child an effect analogous to or identical with itself; and even of these curious coincidences the records of physiology are not wanting. But to gainsay the disturbing influence of emotion on the progress of pregnancy, is to flout a constantly recurring truth.

The mind of the nursing mother may also very speedily derange or contaminate the circulating fluids, the blood, its products, deposits, and secretions. Dr. Underwood adduces an illustration of this truth. A visitor had suddenly dropped dead in the presence of the lady of the house. Immediately after this, even during the intensity of her alarm, she put her infant to the breast. It was very soon seized with a fit, and remained for thirty hours in alternate states of convulsion and coma. I adduce this merely to point to the contamination of the fluids from mental influence.

This emotion may also produce morbid effects on the generative system of a more protracted duration. Dr. Greding, of Waldheim, has recorded the case of a woman whose daughter was killed by the stroke of a tree which the mother had just felled. She became soon after pregnant, and produced a male child, who was born a maniac. He possessed so much strength in his legs and arms that four women could at times with difficulty restrain him. These paroxysms either ended with an indescribable laughter, or else he tore in anger everything near him. We durst not allow him to be alone, otherwise he would get on the benches and tables, and even attempt to climb up the wall. Afterwards, however, *when he began to have teeth,* he fell into a general wasting, and died. I am not prepared to assert the potent influence of paternal senility in procreation, although Breschet and others believe it. We cannot but wonder that even arrest of development does not more frequently occur, when we reflect on the tenuity of structure, and the delicate connexion of the fœtal tissues; the slender filament, for instance, which unites the head and trunk ere the neck is fully formed; and especially when we are aware of the frequent repetition and excess of conjugal endearment, even to the eleventh hour of pregnancy. I do not doubt that this is a frequent cause of abortion.

I may here add the probability of tight-lacing as a cause, for it has been affirmed, by Breschet and Goëlis, that the children of unmarried women, who have attempted to conceal their shape, were the most frequent subjects of effusion. While the fœtus is passing through those progressive changes, each stage of which so beautifully illustrates the ascent in the scale of creation, from the zoophyte to the perfect being, those conditions of comparative deficiency or excess, of atrophy, or hypertrophy, are induced, which constitute the anomalies of monstrosity. Although there may be superficial indications of perfect health, yet the nervous and vascular systems of the fœtus may be so influenced, or the fluids be so tainted by the maternal springs, that the infant may be born either with an intense degree of irritability, or of laxity of vessels, which become the fertile source of disorder, or with that germ of disease that only waits for excitement to induce its development. This constitutes the essence of predisposing cause. Of these tendencies, or predispositions, the strumous diathesis is most prominent; yet even that may be so slightly marked at birth as not even to be suspected. On the onset of cerebral disorder, however, its existence may so weigh with us as to justify an assurance of great peril in our prognosis.

The subject of the tubercular diathesis is of the greatest importance, as it not only predisposes to disorder, but renders that disorder intractable and perilous. When, for instance, encephalitis occurs in a healthy brain, the form is phlegmonous, and it is more amenable to active treatment.

The very frequent discovery of granular tubercle on the pia mater, especially about the base of the brain in fatal cases of effusion, (ninety-five per cent., according to Dr. Hennis Green,) will induce us to believe that it may constitute the essential tendency to hydrencephalic disorder.

In very many of these cases, the tubercular deposit is not confined to the encephalic membranes. The lungs especially, the liver and intestines, the mesenteric and bronchial glands, are often either studied with granular tubercle, or coated with flaky caseous patches. I have often seen the mesenteric glands only exhibit the true strumous degeneration, when there have been no evidences in any other tissues, especially in cases of low remittent fever.

With these important facts before us, when we see associated with cerebral symptoms deficiency of cranial ossification, enlargement of the ends of cylindrical bones, tumid abdomen, and enlarged mesenteric gland, and especially if the condition of several children in a family indicate hereditary or constitutional taint or tendency, we must consider the child intensely disposed to meningitis and effusion; and we cannot be too watchful and prompt in our ministration, or too cautious in our prognosis.

In our consideration of intra-uterine defect or disease, we must commence at the very lowest point of animal existence, the mere ganglionic vitality of the zoophyte, which is, indeed, in utero, the early fœtal condition.

It is true that there is life in that being in which the spinal marrow itself is wanting, but the amyelous fœtus ceases to exist at the moment of its birth. If the spinal medulla be prolonged to the marginal line of the occipital foramen, there may be a brief respiratory or extra-uterine vitality. The evolution of heat, the respiratory and excretory functions, and the excito-motory action of secretion, may be, to a certain degree, duly performed, they being all, at least for a time, independent of cerebral influence. Under this condition the child may survive for many hours, even for several days. Ollivier adduces the case of an anencephalous child that lived two hours; Dr. Marshall Hall refers to one which survived fifty-five hours; Lallemand, to another which lived three days; and in Mr. Lawrence's paper, in the *Medico-Chirurgical Transactions,* is recorded the case of a child that survived five days, in which there was not a particle of cerebrum or cerebellum. Cruveilhier relates a case analogous to this, in which there was a little nodule on the basilar groove of the occipital bone, about the extent of cerebral development in the asteria, or star-fish.

In the case recorded by Mr. Burrows, in the *Medico-Chirurgical Transactions,* there were several interesting phenomena. There was *no evacuation,* although the ganglionic and spinal systems seemed perfect. But the excito-motory actions (at that period little understood) were very decided. The least peripheral irritation excited spasm and convulsion, and pressure on the brain produced a sort of electric shock, although the child was usually torpid and passive.

In this case there was a general absence of cranial bones, merely a small portion of the occipital existing, the cerebrum and cerebellum appearing like a hernia of a mulberry colour. The child, however, lived, with irregular respiration, six days, having no evacuation, and taking no food.

Dr. Bright records the case of a child two months old, from the cranium of which Dr. Remmett, of Plymouth, drew, by five operations, eighty ounces of fluid; and when the child died, two quarts of fluid filled the cranial cavity. The medulla oblongata was entire, and there was a small medullary mass lying behind the orbits. Dr. Cheyne would probably have affirmed that this fluid was compensation for the want of brain; Morgagni, that the fluid destroyed the brain by compression.

With these melancholy states of complete cerebral atrophy, we may, I think, believe (in opposition to the arguments of Lallemand) that sensation is completely absent. Were it not for this conviction, the accoucheur would often feel very deep regret during his performance of craniotomy, especially if the child should move and cry subsequent to birth.

Some years since, with the concurrence of my friend Dr. Davis, I evacuated the encephalon of a very large and well-formed child in the city. The brain and its vessels were completely broken down, and rather profuse hæmorrhage had supervened; yet there were free movements of the limbs; respiration commenced, and a faint cry was heard for many minutes subsequent to its expulsion. Mr. Sweatman, in a letter to Sir Charles Bell, relates a case somewhat analogous, in which motion of the lower limbs continued for half an hour subsequent to birth.

In these cases of acephalous monstrosity, the integuments are usually collapsed and puckered, consisting partly of cutaneous tissue, and partly of membrane; sometimes, though mis-shapen, they are fully formed, and contain a membranous bag of fluid — a sort of cerebral vesicle, the brain being either altogether wanting, or so diminutive as to be compressed on the base of the skull. The condition of these anencephali is identical with spina bifida, differing in position merely. I do not remember a case, however, in which I have observed the serum limpid. In the sheep it is usually so. A case of this kind is recorded, in which, after the operation of trephining, the compressed brain expanded, (of course from the renewed circulation,) and filled the cranial cavity.

A still-born child was brought to me, some time since, by a midwife, in which the cleft or ossific deficiency was in the occipital bone. The frontal was very diminutive, and receding from the orbitar ridge—the *katzenköpfe* of the German pathologists. Through this cleft a mass, composed of medullary pulp macerated in fluid, appeared to have been gradually forced, so as to distend the membranes and integuments into a separate cyst, nearly as large as the cranium itself, which lay along the spine like the ears of a hare, weighing down the cranium and face, so that they almost formed a continuous line with the vertebral column. The form of face was that of abject idiocy.

A preparation of a somewhat similar being was presented to Guy's Hospital Museum by my friend Mr. Hargraves, of Tonbridge Wells. In another child, which survived two days, with the very faintest marks of vitality, the encephalic mass of medulla and fluid were contained in a pouch of natural integument, which was continued from the edges of a very large anterior fontanel, which soon assumed a gangrenous appearance in patches. There were very slight excretions involuntarily passed, but the excito-motory influence was insufficient to induce the infant to suck.

In all the cases of this state which I have seen, various degrees of paresis were invariably observed. In the case related by Sir Charles Bell, in his work on the nervous system, there were occipital and vertebral deficiency; the bag containing the medullary pulp being broken during parturition. In this child, motion was observed for three hours, and very slight respiration for about seven hours; but these were the only prominent indications of vitality. In these and analogous cases, the disorganization of the cerebral medulla rendered the beings incapable of continued vitality, putting them at once out of the pale of our association.

Some of these medullary softenings seem to be antecedent to effusion, not the consequence of maceration; as we occasionally find degrees of congenital ramolissement without a drop of serum. They are forms of atrophy dependent on anæmia or deficient nutrition, and not the result of inflammatory action.

Billard, Copland, Andral, and other pathologists, have recorded cases in which the cranium and even the whole spinal canal were filled with a disorganized pulp, which was observed, by Billard especially, to possess an odour of sulphureted hydrogen. The external mark of these children evidently indicates struma, being often combined, not only with defect of cranial ossification, but with downy skin, excess of capillary growth, hypertrophied extremities of cylindrical bones, *clubbed* fingers and toes, and glandular enlargement.

In some cases of congenital hydrocephalus, the membrane only is protruded—hydrencephalocele meningea; in others, there is a more decided centrifugal tendency of the secreted fluid, unravelling or spreading out the convolutions of the brain by an immense volume of water—dropsy of the encephalon—or distending, perhaps, one ventricular cavity, and producing a hernia of the encephalon; the condition of the encephalic mass being so far normal as that vitality may be protracted to an indefinite period. Otto believed that in all the cause was effusion, and not the growth of brain.

Of the first class of cases, of many of which I have preserved correct drawings, several have been presented to me at the Royal Infirmary; others have perished in their birth, or the fluid has been discharged ere they could be born. The children I have seen have been generally wan, pallid, and unsymmetrical in their growth. In the latter cases, the disorganization, probably, sometimes depends on disease, or fault of the placenta, or the cord.

A child was brought to me nearly a year old, the head of which resembled that of some nondescript animal rather than of a human being; the face was most diminutive, occupying no more than one-twentieth portion of the sphere of the cranium. The measurement round the forehead and occiput was twenty-two inches; from the chin over the vertex to the occiput, twenty-one inches. The sutures were united by membrane, and there was deficiency of ossification in the centre of the bones. The symptoms were, intolerance of light, (the pupil, however, being dilated;) diarrhœa, marasmus, convulsion, and excessive moaning. Yet this child survived twelve months. When it died, the dura mater was found firmly adherent to the cranium; the pia mater extremely vascular, and three and a half pounds of fluid in the subarachnoid tissue. The extremity of this case forbade interference.

When, however, the general symptoms are less severe, and a superior degree of consciousness is apparent, these unhappy beings are not without the possibility of remedy. I was requested to see a child about three months old, in Green Walk, Christchurch. Its cranium was enormously distended, and the superficial veins immensely congested. These were all the prominent symptoms of effusion. I punctured the cyst four times, evacuating a straw-coloured serum; the operation being invariably followed by a relief of the convulsive action. The child lived one year. The cerebrum, or rather, the two lateral and third ventricles, were dilated into one enormous bag, or shell, the pia mater being opaline, and thickened and traversed with turgid bloodvessels of all sizes. Within this encephalic mass, we could discern only the following tissues:—The choroid plexus turgid; the thalami immensely hypertrophied; the anterior commissure; and the two optic nerves. The cerebellum was perfect.

I was requested to visit an emaciated child with a very large head, which had all the usual signs of chronic hydrocephalus. There was one *peculiar* symptom: it would squeeze its eyelids together very forcibly, as if in extreme pain, and scream, *tears* flowing copiously. In a short time it would waddle across the room, and even try to speak. I punctured the membranes three times, the child subsequently becoming fat; it died at three years of age. Two pints and a half of fluid were found in the ventricles dipping down into the third; the septum lucidum was formed into bands; the optic nerves lost in the corpora striata; a large medullary band connecting one corpus with the other.

Another child was brought to me with a most diminutive face, and the cranium enormously distended, the sutures being all united by membrane. There were, strabismus, dilated pupils, and spasmodic twitchings; sometimes *violent* convulsion and opisthotonos. The bowels were sometimes confined, at others, relaxed; the child sucked ravenously, yet wasted progressively To relieve distention, more than with a hope of remedy, I punctured the membranes with a small trochar, and drew off four ounces of limpid serum. The convulsion very soon subsided, and the child rallied. A week after, I took away ten ounces, the child smiling soon after the operation. A third, fourth, and fifth introduction of the trochar were followed by very large evacuations of fluid, the bones and integuments directly *falling loosely together.* Yet though I used no compression of bandage, the child never suffered syncope: it was thus saved from death. When I last saw it, it had lived three years, and seemed to enjoy its existence. It may yet be alive, but of course it will be during its whole life an idiot.

I am aware that it will become a question whether operation in these cases be justifiable when we reflect on their unpropitious result, and remember also that life may not only be preserved, but even enjoyed, for the space of thirty years, without the slightest remedial interference.

Mr. Earle has related the case of a girl, twelve years old, then living in St. James's Workhouse, in the full enjoyment of existence, who was born with a large diaphanous tumour on the occiput; and we all remember the very interesting case of Cardinal, who died at the age of thirty-two, in Guy's Hospital. In his cranium, not only was there a cerebrum weighing two pounds four ounces, but a pint of serum in the ventricles, and nine pints between the dura mater and arachnoid. This man was harmless and cheerful, very fond of being noticed, and especially delighted by the assurance that his head was growing larger. If we believe, however, that it is our duty to preserve life under any condition, we should be guarded in our decision regarding operation by the prospect of relieving severity of symptoms, (as we would in the analogous diseases of ascites and hydrothorax,) especially those of convulsion. Nature herself seems occasionally to point to this. Dr. Bacon (*Medico-Chirurgical Transactions*) relates a case in which the brain and arachnoid burst, the escape of fluid affording much relief to the child. In the second case which I relate, dissolution was imminent; and healthy life, as far, at least, as the organic functions were concerned, was for a certain period insured. The success of the operation will, however, depend essentially on the degree of cerebral disorganization.

In some of those instances in which the symptoms are slight, and the patient survives for a period of years, the fontanels and sutures are very gradually closed, the fluid ceasing to be formed before complete ossification. If during the progress of effusion the ossific process be early completed, the type of symptoms immediately changes, and either the acute form ensues, or the fatal effects of compression are soon witnessed. In the case related by Dr. Baillie, ossification was not complete until the subject was seven years old; the sutures then began suddenly to separate, and continued to do so until death. One pint of fluid was found in the ventricles. In some cases, I may add, there is deficiency even of half the cranium; one portion being bone, the other, membrane and integument.

Now the acephali resemble so much the embryo of even the sixth week, that it is probable about that time, or just before it, the development has been arrested. The head of such an embryo is transparent—as Tiedemann terms it a cerebral vesicle. At the seventh and eighth week, the membranes and cerebral pulp are first discernible. We may believe, therefore, that some defect of nutrition, or it may be, some mental influence, then occurred, and from that moment the cerebral growth was negatived. So early after impregnation and deposition of the ovum may the character and destiny of the creature be decided!

It is probable that in the early stage of other of these hydrencephali, the secretion of normal fluid is in excess, in consequence of the general laxity of fibre, medullary as well as vascular, and that systemic debility which reduces the tonicity of the exhalent, so that the fluid drops away, as it were, from its mouth, while it renders the absorbent also inapt for its duty; a want of balance instantly ensues; effusion of course accumulates, either into cavities or between membranes, or within the convolutions of the brain.

I believe fœtal effusion is sometimes associated with a dropsical state of the mother, and also with dropsy of the amnion membrane. These cases illustrate, probably, one form of chronic effusion, the result of laxity of fibre; the different parts of the encephalon, although dislocated, still being nearly or quite perfect. Atrophy, or arrest of development, may, however, commence, from maternal influence or organic defect, (the proximate cause probably existing in the pia matral vessels,) just as the spinal marrow or medulla oblongata were completed, or at any other progressive stage of encephalic development, that portion which *is* found being normal or healthy. If the arrest be early, we have still effusion as a compensation; if at a later period, the brain may be either perfect in its parts, though deficient in size, (general atrophy,) or deficient in particular lobes or structures, (especial atrophy or non-development,) the want supplied by effusion or by the cranium itself being kept proportioned to its contained mass. The atrophied brain is stringy, almost fibrous, from absorption of the more pulpy tissue. The brain usually weighs about two pounds when the subject of atrophy is nearly two years old. If the anterior lobes be in defect, we observe, of course, the shelving forehead so characteristic of idiocy, and this atrophy may even be to a degree progressive; for the brain of such an idiot lying fallow, one stimulus of growth is wanting, and the organ of mind will *dwindle;* thus may we account for the *increase* of fatuity even in congenital idiots, when we might, perhaps, rather anticipate some *expansion* of intellect.

In the misshapen or one-sided cranium, I believe the lobes of the cerebrum will often be found disproportionate; and it would be a curious question with those who favour the notion of *duality,* how far this want of relative development, and, consequently, of energy, might explain their hypothesis. There may also be arrest or absence of various other structures, as the corpus callosum, corpora striata and thalami, or of the small glandular appendages, the pineal and pituitary bodies; indeed, Otto so often found them wanting in idiots that he might almost have become a proselyte of Descartes, and located the soul itself in the pineal gland. I am not ready to assert the pathological evidences of these defects, although they must, of course, refer to the structures thus involved. The *imperfect development of convolution* will be found sometimes associated with the state of idiocy; for the cerebrum has been found in some of these children merely one medullary mass, the hemispherical ganglion being spread over it as a thin grey film. Development of convolution, however, is naturally in abeyance during the first year; but then we still perceive the purplish hue of the ganglion in *striæ* below the medulla.

Of the second class of cases, hernia cerebri, I have seen many instances; in these, as in spina bifida, there is essentially ossific defect.

A male child, ten weeks old, was brought to me with a diaphanous tumour on the left side of the head. The labour was natural, and the infant seemed for a few days to thrive, when symptoms of remittent fever supervened; aphthous vesicles studded the mouth; diarrhœa of sour green fluid evacuations occurred; and emaciation commenced, although the infant still sucked regularly, and frequently retained the milk. Astringents &c. checked the diarrhœa; the tumour increased, and we observed the usual symptoms of cerebral disorder, especially those of slight compression. The tumour now measured upwards of six inches in circumference. During the space of nine days it was punctured thrice with a grooved needle, and about twelve ounces of fluid drawn off. Neither syncope nor any other unfavourable sequence occurred until the last puncture, when the infant became slightly convulsed, and expired on the following day, the tenth from the first operation. The sutures were generally united by thin cartilage, and there were a few ossa triquetra. The left parietal bone was nearly divided by a cleft of two inches in length and half an inch in width. Around the cleft, the pericranium was lined with the protruded dura mater; the edges of the cleft were cartilaginous; fibrous bands crossed the cleft at both ends, forming a firm connexion. On removing the parietal bone, the ventricle which formed the cavity of the tumour was exposed, its membrane being lined by a layer of lymph, in which minute vessels ramified; the cerebral mass was softened, and there were several small varicose tumours on the plexus choroides. I believe in this case the formative process of the cerebral lobes was arrested, the fluid by compression preventing the completion of the ventricular ceiling, and also the centre of the parietal bone. Before this arrest, the cartilaginous nidus of bone partially, and the integument had been already produced according to the progressive order of the cranial development. The ramollissement and deposition of lymph were probably the result of inflammatory action induced by the puncture, the formation of the lymph being, in fact, the effort of nature to restore.

I may add that I have seen clefts in parietal bones without protrusion; in these cases, probably, effusion had taken place, and subsequent absorption.

A case analogous to the above is related in the *Medico-Chirurgical Transactions* by Mr. Earle, the diaphanous tumour being over a cleft in the occipital bone. The operation of paracentesis was repeated nine times with various instruments, the child surviving nearly two months from the first operation.

These fluid herniæ sometimes take very eccentric courses. In one case related by Dr. Creutzwieser, of Königsberg, of a person twenty-six years old, through a cleft at the outer angle of the os frontis, the fluid formed a large pendulous bag on the cheek. Breschet has recorded a case in which the brain and medulla oblongata were protruded into the spinal canal. The fluid has also escaped sometimes from the lowest portion of the sacrum by the sacro-sciatic ligament, and become lodged under the gluteus maximus.

We must remember to distinguish these cases of encephalocele from the cysts sometimes found on the side of the head of the infant, which contains a glairy fluid, and which, in consequence of the external lamina of the bone being deficient, while the internal is perfect, impart the conviction that there is a communication with the membranes of the brain.

Combined with these conditions of disorganization or arrest of development, and consequent diminished intellect, we observe generally an exuberance of the ganglionic system, especially of the sympathetic. The character of the being thus defective is, of course, that of idiocy in its varied degrees, from stupidity to absolute and degraded fatuity—from the creature who, always a child, is ever laughing at nothing, or charmed with bright colours or light, and, with its mouth open, employs its time in catching flies, to that supreme idiot, the congenital cretin of the Alpine valleys, and other intramontane districts.

We are often consulted on the cases of these Davy Gellatlys in swaddling-clothes, of whom such is often the degree of animal enjoyment, that even the anxious mother will almost cease to grieve, and will smile on her idiot baby almost as she would on her pet lamb. To the stranger the idiot appears such a mere laughing or dancing vegetable, that pity soon yields to a feeling of curiosity or mirth.

The contour of the melancholy cretin is in just keeping with its imperfections. The enlarged cranium lops heavily on the shoulders—the face is marked by dull, listless apathy—the creature is often deaf and dumb—thought and sensation seem to be banished, save the one passion of sexual instinct; indeed, the insensibility is sometimes so extreme, that the thing will not seem to feel even while his flesh is burning. The cretin is from four to five feet high, his chin often cadaverous and flabby, and studded with livid eruption—the head immensely out of proportion—the eyes blear and squinting—the mouth slavering—the limbs weak and crooked. Like the *Stulbings* of Swift, the senses are imperfect, the expression being either that of a fool or a satyr.

In all the endemic cases, goitre is more or less developed. Cretinism is not always, however, congenital or hereditary, but the same atmospheric influence will, I believe, impart the malady to the fœtus through the maternal system, and induce the *endemic degeneracy* (as Maffei calls it) about the period of the first dentition.

The morbid anatomy of cretinism is varied. Even from the elaborate essay of Greding, we must fail in pointing to any especial sign. The brain is sometimes an atrophy, or it may consist of white ramollissement, or a bluish jelly, or it may be very firm in texture; but it is never *healthy* neurine, the deviations being effusion, opaline membranes, hydatids, scirrhus, and a diminution of convolutions.

From these structural depravities there must be a degree of asthenia of vital function. Yet slight cases of true cretinism may, by persevering and judicious education, be relieved and even cured. Among existing proofs of this, Dr. Odet, who was himself a cretin, has written a very sensible essay on the woful malady.

It is not only in its intra-uterine state that predispositions are established. In its passage into the breathing world, in laborious labour, or through a pelvis deficient in capacity, the head of the child is often subject to mechanical compression, the occipital bone being frequently driven beneath one of the parietals; this may either cause immediate destruction of vitality, or induce some chronic action in the brain, that may become the cause of susceptibility of subsequent disorder, or it may induce the condition of hernia cerebri.

When the infant is still born, we may believe that the brain is the subject either of congestion or extravasation, and on this discrimination depends our success. Where the state is that of congestion, and compression has not long been continued, artificial respiration will usually be successful. On the contrary, if the compression be protracted or very forcible, we may find one of two conditions: either a state of irremediable asphyxia, or one of extravasation. We cannot, however easily discriminate, as there are no symptoms: the child is still, and has ceased to breathe. Even if this condition be induced by pressure on the funis, still there is a poisoned fluid that oppresses the brain and the nutrient vessels of the heart.

It is essential, therefore, speedily to excite the heart by its healthy stimulus, and this cannot be done without respiration. The child should first be put into the warm bath, or sponged with warm water, and then very cold water should be dashed on the face and thorax of the child, for awhile exposed to the atmosphere. This will usually succeed. I have attended a lady in Baker-street, whose parturition is always laborious, and who bears very large children, two of which were born in a state of asphyxia, perfectly passive and inanimate. By exciting the trifacial and spinal nerves, by cold douche, and by assisting thoracic action, directly a gasp was observed, both these children were restored.

In cases more extreme, inhalation should be employed, the accoucheur inhaling as much atmospheric air as possible, and then inflating the lungs through muslin stretched over the mouth, the trachea being pressed against the spine. Enemata of warm gruel may also be employed, and, in extremity, galvanic shock may be passed through the thorax and spine.

It has been observed by Dr. Marshall Hall, that an infant is liable to lapse into secondary asphyxia, when seemingly safely recovered. I have not seen this, but it will be wise to watch against such a relapse; if it occur, stimulating enemata and a few drops of brandy may be employed.

When, in consequence of extreme pressure on a soft brain, blood even has been effused into the cerebral medulla, it may not always prove fatal, for cysts have been discovered from which coagula have evidently been absorbed. When the effusion is slight, and in the subarachnoid tissue, there may be hope of this absorption, but when it is in the ventricles it is usually more copious, and fatality soon takes place.

Directly it is born, the child is an independent being, is subject to a variety of external influences, and impelled to a variety of new actions. Its passive, dependent, and unintellectual vitality is past, and the sympathies of the brain are displayed; for although mental phenomena are not directly manifested, yet it is immediately conscious of impression on the senses. It is, moreover, called on to preside over, regulate, or assist many of the most important organic functions. It is not, however, yet predisposed to acute excitement. From this cause, combined with the subsequent processes of ossification and dentition, it becomes sensible of every external influence.

The general fatality from primary dentition is probably in the proportion of about 1 in 15. Erethism in some degree attends its progress, passing off slightly in the healthy child, especially if drivelling, slight cutaneous eruption, or even occasional mild diarrhœa supervene —within certain limits, a natural relief. There is therefore now an excess of determination of blood to the head, as indeed is clearly exemplified by the flushing of the cheeks and the frequent establishment of eruptions. "*Ubi irritatio ibi adfluxus.*"

We must remember that the rush of blood to the nutrient vessels of the teeth is through the same *trunk* which leads to the brain, the branches of which will usually all partake of the increased action; the first contact of cold air induces an indirect action on the brain,—the first ray of light on the retina, the first undulation of air on the internal ear impart direct sensation to the brain, and a consequent excitement, which may become intensely accumulated. It depends on idiosyncrasy, or condition of system, whether this excitement pass off, leaving the brain quiescent, or that state is induced which constitutes irritation of the brain. This is a subject, indeed, of very high importance, for it is to this source that many of the morbid changes of the brain in after life are to be traced.

And how numerous and perilous the cerebral diseases of children prove is learned at a glance from our statistical reports. Even the *specified* cases of hydrocephalus and encephalitis form the larger mean number, except convulsions, unless measles, and scarlatina, and pertussis are prevalent. But, if we believe the truth that in hooping-cough, scarlatina, measles, and teething, which are separately recorded, cerebral symptoms, in an immense majority of cases, precede dissolution, and that as many of the cases of convulsion proceed from cerebral disorder, as those which are traced to other sources, we may term disease of the encephalon the great destroyer of the infantile population.

This condition of excitement, the antithesis to a state of slumber or repose, seems so natural a consequence of the new existence, that it is not strange it should be disregarded even by the pathologist. Repose, however, is the life itself of the infant, and if this erethism of the brain continue, there may easily ensue an undue lighting up of action, and the child may be brought to a state of real disorder, its exciting causes being intense light, loud or continued noise, suppressed perspiration, repelled eruptions, coup de soleil, concussion; the internal causes, retention of meconium, and acrid or crude ingesta.

The great characteristic of convulsive action, to which from the first moment of life the infant is so prone, and which I believe may often be referred to laborious or instrumental parturition, or other cranial compression, may now also be increased, perhaps by the struggle which attends the establishment of the double circulation in its suddenly expanding the hitherto collapsed lung. These are more often the immediate causes, indeed, of that spasmodic condition "trismus nascentium," than the ulceration or division of the cord, so much insisted on by Leroy, Colles, and others, who would yet, I suppose, be wonderstruck at the recommendation of a collegiate professor to leave the placenta pendant to the abdomen of the infant until it dropped from the funis.

### CEREBRAL ERETHISM.

The character of cerebral erethism is restlessness, starting on very slight impressions, rolling of the head on the arm or pillow, an insidious smile around the angle of the lip. The expression of complaint does not yet exceed that of simple *fretting*, occasionally, however, lapsing into a whining cry, which is attended in older children by a copious flow of tears. The pupil is usually somewhat contracted, but in some cases perfectly natural; there is often a slight degree of carpopædal spasm, and the head is frequently thrown back for a moment, an indication of cerebro-spinal participation.

These symptoms may very quickly subside when the system is healthy, but in strumous or cachectic infants they are very likely to increase, especially if the ingesta be not duly regulated. After awhile the vessels of the brain become turgid; there is slight strabismus and regularly remittent febricula; the tongue is white, and there is languor and lassitude from the rapid exhaustion of infantile irritability.

In a more advanced period of infancy, erethism and its concomitants may be increased by more important causes. The process of dentition, if it be combined with excess of intestinal irritation, or hepatic derangement, or if it be synchronous with scarlatina, rubeola, or pertussis, is one of peril. The combination of dentition and of extreme derangement of the intestinal canal are the constant predisposition and excitement of idiopathic convulsion, or *inward fits* in infants; a spastic rigidity of the limbs, sometimes so often recurring and passing off almost without observation or anxiety; unless the cerebro-spinal axis becomes involved, and the convulsive twitchings are combined with lividity of skin, coldness, insensibility, and slight opisthotonos.

Now, the mucous membrane will often, under this excitement, assume a vicarious action, and by its profusion relieve the more important tissue. It is of deep and vital importance to decide as to the propriety of checking this diarrhœa, and thus throwing back the action on a membrane such as the arachnoid, *having no natural outlet.* To learn this valuable secret, the etiology of the alternations of cerebral and intestinal derangement is of the deepest importance, for it is a closed volume to the superficial pathologist, yet on our decision as to cause and effect hangs the very thread of life. The head and alimentary canal are so intimately associated in sympathy, that their pathology in the infantile state is one constant reagency. Dentition deranges the alimentary canal, and it is gastric or intestinal irritation, especially in the unhealthy system, that renders teething perilous.

If attended to early, and carefully watched, all these formidable symptoms may usually be averted, even after the twentieth recurrence. About two years ago the infant of Mr. L——, a merchant in the city, was the subject of at least a score of these attacks, and on several of these occasions the child appeared lifeless, its state hopeless. Although the pupils were mere points, yet, as the *fontanels were depressed,* I abstained from leeching. The warm bath, free incision of the gums, ether lotion, a line of vesication, castor oil, warm water enemata, and the tincture of assafœtida, (their combination properly regulated,) invariably relieved the child from its peril, and it is now a very healthy boy. These cases are often referred to effusion, and a very glad surprise is felt and expressed at the prosperous result. But the combination of these causes at this critical period is sometimes followed by effects far more sudden and perilous. Indeed, when we consider the intimate intercommunications of the nervous system, we cannot wonder at the pathology of its sympathies.

It is under these conditions that a very formidable disorder is often developed; sudden in its onset, insidious in its nature, often perilous, and fatal in its termination.

### STRIDULOUS CONVULSION.

When Dr. Clarke first alluded to the "peculiar form of convulsion in children" which has since obtained the varied designations of chronic and cerebral croup, crowing convulsion, crouplike convulsion, laryngismus stridulus, acute asthma, laryngeal asthma, (perhaps the preferable term would be stridulous convulsion,) he fell into some error of etiology and pathogeny. When he affirmed that the brain was originally affected, he probably alluded to *predisposition.* But Clarke wrote before the researches of Marshall Hall had reconciled us to the belief that spasmodic action of the laryngeal muscles might arise from spinal irritation alone. I believe that this convulsion may take place in a few instances without warning; but these are exceptions to the rule, and occur usually after a full meal, or when the child awakes startled from its sleep, especially if it has been exhausted, and its head has laid on a low pillow. But in watching suspected children carefully, we shall constantly observe, it may be *slight*, premonitory symptoms; such as twitching, smiling, the infant fixing often eagerly on the nipple, and then rejecting it without sucking; the rolling of its head, and fretting, and startling on the slightest alarm, the eye turned inwards, often out of slight distortion of feature and slight coma. There may be a livid, dry state of the lips, some degree of dysuria, and absence of biliary flow, the state of bowels being in one extreme or the other. With many of these symptoms, if slight, a sudden crow may be the first cause of attention or alarm on the part of the mother. Of this alarm the child evidently partakes, for it screams with the most anxious expression, as if to implore relief.

In very slight cases a change of position, or even patting on the back, will immediately relieve; a short inspiration and deep expiration, and the child will for a time be well. If the spasm continue or recur, the face will become more livid, the brow frowning, the distress greater; opisthotonos may be so intense that the body will be a rigid bow. Air is drawn in with extreme effort; there is then for a time a shrill squeak, until the child dies in the convulsion.

I believe Dr. Marshall Hall correct when he affirms the remote causes to be irritation of the extreme fibrillæ of the nerves, inducing reflex action, and thus spasmodically affecting the respiratory valves; the various excitements of the pneumogastric, the trifacial, or spinal, all tending to one focus, the constrictor laryngis.

There has been much discrepancy of opinion, and several hypotheses have been adduced regarding this affection. It is probable that isolated cases may occur in which encroachment of a gland on the recurrent nerve may cause irritation and spasm, but this cannot be firm or complete compression, which would produce paralysis rather than spasm, as we find in the case of tumours on the neurilema. If Dr. Ley's notion were always correct, we should not find the frequent remission or recurrence, nor the sudden relief and recovery with which we are so often gratified. I believe, in the majority of cases, the malady is excited through the spinal system, but excitement of the cerebrum primarily, and from mental influence alone, especially sudden alarm or fright, will often induce it in children predisposed.

It is probable that gastric irritation, especially when aided by malaria, is more influential than mere dentition; for when the bowels are duly regulated and fresh air inhaled, we very rarely witness this affection.

This truth will point to those valuable prophylactics, regulation of diet, pure air, and exercise.

On the recurrence of the paroxysm, however, the most prompt treatment is requisite, as asphyxia is imminent. Free and deep scarification of the gums should be instantly adopted, chiefly to deplete the vessels, especially if the mouth be hot and dry; and this whether the tooth be near the surface or not, as it is in the *breeding*, more than in the cutting of teeth that the irritation occurs. The infant must not, after this, be put directly to the breast, as it may swallow blood, which coagulating, may still more irritate the stomach. If subsequent symptoms of meningitis occur, leeches should be applied, or a line of vesication drawn behind an ear, or along the sagittal suture, in some cases followed by the dressing of mercurial ointment once or twice. Irritation of the fauces with a feather, or a mild emetic, castor oil, or enemata, may be adopted, according to the judgment of the medical attendant.

Thus much in parenthesis regarding laryngeal spasm.

If it be not interrupted, the fever of irritation will now assume a more decided type, and the remittent form, the surface of the body being hot and cold by turns. The head is constantly lopped on one or the other side, and the child constantly sinks for a few minutes into a comatose state, often mistaken for slumber. The body becomes rigid, and the teeth are firmly closed; the muscular irritation is increased; sometimes there is opisthotonos and convulsion; there is intolerance of light, the strabismus is increased, and the eyeball is rolled in its socket; the pulse becomes more laboured and oppressed, the respiration irregular, and often sobbing; the urinary secretion is suppressed, and the intestinal mucus being scanty, the bowels are torpid and confined. Hyperæmia and congestion of the cerebral vessels has now supervened, and we are in peril of many morbid results. One of these may be immediate; there is a remora of dark blood in the sinuses and meningeal vessels, and the capillaries become gorged. Thus there is not only compression, but a poison on the brain; and the organic functions are all in fault from defect of their proper and healthy excitement; and the infant may in this very early stage of the disorder die in asphyxia. In very plethoric (what are termed *fine*) children, cerebral congestion will often occur: of this we may be confident when eclampsia and coma, and in older children, delirium, supervene. The effect of this detention of carbonized blood in the brain closely resembles epilepsy, save in the absence of foaming.

It is in this simple state of congestion—so often misnamed hydrocephalus, and in which the seeming severity of symptoms so often misleads—that we are called on to exert our most judicious reflections. It is equally essential to support the power of the languid child, and to prevent exhaustion, as to subdue excess of action in the more robust.

The first indication will, of course, be to relieve the turgid state of the meningeal vessels by equalizing the circulation. The warm or tepid bath should be invariably and quickly resorted to for five or ten minutes, and the child be then wiped dry with warm flannel. If the bowels be torpid, castor-oil should be given, or, that which is preferable, an enema of thin gruel and salt, with a few drops of oil of turpentine. Folded rags dipped in ether and water should be laid along the sagittal suture, and a few drops of tincture of assafœtida given in warm water. If the child be plethoric, depletion may at first be freely adopted, especially in acute cases; it may be essential to open the jugular vein; and a large dose of calomel will often be very useful in exciting action of the liver—this viscus in fatal cases is usually found gorged. Among the morbid appearances in the encephalon, there will often be not only congestion of the sinuses and veins, but firm coagula of loose blood, branching even into the veins themselves—a product easily distinguished from those of pus and lymph.

But the analogous symptoms which occur, with very slight variation of degree, from exhaustion, and from plethora, as well also during profuse diarrhœa as from obstinate constipation, render it our duty to study diathesis with great care.

In the strumous or languid system, from this impeded circulation, and other causes, to which I have before alluded—a want of balance ensues between the secernents and exhalants, the result of which will be the passive or insidious form of hydrencephalus.

Depletion should therefore, in such children, be cautiously adopted, the aperient especially being so mild as to avoid the slightest risk of hypercatharsis. I have seen children lost by active treatment, in which slight meningeal congestion and general anemia, especially on the surface, were the only abnormal appearances discovered.

It is in this strumous child especially that we witness the fatal effects of free depletion. The symptoms consequent on this error may closely resemble those which it was our aim to combat. It may be said, that by free depletion, even in advanced life, we may relieve, or even restore for a time, deranged intellect, yet it is proved that irritability and tendency to effusion will more rapidly increase chronic hydrocephalus. In experiments on those animals which have been bled largely, very profuse effusion was discovered at the base of the brain.

It is this excess of depletion, then, which may induce that train of anomalous and obscure symptoms, yet so nearly resembling those of effusion, that have induced Dr. Hall to term the state *hydrocephaloid*

*disease.* This perplexing condition, however, may be, indeed, and often is, induced by profuse diarrhœa or hypercatharsis, and by very protracted and inefficient lactation.

A mere defect of nutrition, which may induce fatal marasmus, is not marked by so prominent a set of symptoms—they are more gradual; yet death may be immediately preceded by convulsion, thus imparting more or less resemblance. The primary symptoms of all are irritability, languor, torpor, coma, the first and third degrees being the sensitive and torpid erethism of Dr. Nicholl. From the commencement, the child becomes more and more pallid, the extremities cold, the lids droop, the pulse is laboured and thready; sometimes there is a transient throbbing, similar to the hæmorrhagic effort; the child sobs at intervals, with some slight crepitation in its breathing, towards the close of the disorders; the fontanel is usually deeply sunk.

Even in this prostrate state, the excito-motory influence, as Dr. Hall has observed, may be very prominent; the tickling of the sole often causing the child to lift its foot with a jerk.

The three most important symptoms which should influence our treatment of these insidious conditions are the hue of skin, the state of pulse, and, above all, the depression of the fontanel. If we regard or watch these attentively, we shall not, I believe, fall readily into those errors of diagnosis so often committed. Extensive effusion has been too often discovered when not suspected; and perfect anæmia has existed where meningitis or extreme hydrocephalus was affirmed.

Treatment should of course be adapted to condition and causes. Diarrhœa should be *checked* by cretaceous remedies; prepared chalk with or without aromatic confection, or a few drops of aromatic spirit of ammonia, should be given; and in extreme cases, a minim or two of the *black drop*—the best opiate for children.

If the child be *spoon-fed*, or the subject of protracted lactation, it would be eligible to procure a young healthy breast, for a time, at least, until the symptoms are changed or removed. Warm water sponges should be applied to the abdomen. After excess of hæmorrhage, spirit of ammonia should be administered in warm water; and if the child lie in prostrate stupor, brandy may also be given in warm water, the child being reclined in a semi-recumbent position. In more protracted lassitude and debility, the solution of camphor in fluid magnesia (of Sir James Murray) will often be very useful.

### ENCEPHALITIS.

### FIRST STAGE — SENSIBILITY IN EXCESS.

If the state of cerebral congestion be not relieved, another condition may be speedily induced. The onset of inflammatory action in the encephalon will usually be a rigor and a flush, the effect of the struggle of reaction on the remora of the blood. The brows are firmly knit, the head is tossed from one side to the other, often rolled half round; there is an expression of great anxiety, and the least noise alarms the child—indeed, every sense is morbidly sensitive, even to the state of hyperæsthesia of the skin. In older children, the voice and look of a stranger are subjects of alarm. At this period, the characteristic cry is a shrill slight scream; the symptoms are chiefly cerebral. The onset of fever is soon indicated by white tongue, hot mouth, thirst, flushing of the face, red conjunctiva, and rapid and hard pulse. There is often constant strabismus, the pupil being a mere point, and often out of sight under the lid. At this period, the sympathetic symptoms supervene; the stomach begins to reject its contents; the child often pukes and vomits milk the instant it is swallowed. There is often some spasmodic respiration, sometimes marked by stridulous inspiration. The pulse is now extremely rapid; Dr. Whytt counted 210. It then becomes throbbing, especially the beat of the carotid; the fontanel, even in sleep, rising and falling with much force. The excito-motory influence is now more decidedly evinced; the twitching of the limbs is violent, and there is firm carpopædal spasm; and as the cerebro-spinal axis becomes involved, opisthotonos will be violent and complete. It is rare that the peristaltic action is not altogether suspended. There is often dry, irritative cough, which may, indeed, create some error of diagnosis. There is often much difficulty in swallowing, the fluid returning through the nose. These symptoms mark the dry, inflammatory stage, as it may be termed; and I believe they do not essentially differ whether the medullary or membranous structures, or both, be involved. They are, however, more severe when the arachnoid, and especially the basilar membranes are affected; and in a child of two or three years old, in which the intellect is somewhat developed, there is superadded the more psychical symptoms of ideal excitement, and more confirmed delirium, the special senses being deranged according to the *seat* of morbid action. There is spasm and distortion of the face, and screaming on any movement of the limbs; the heat of skin is almost burning, and the *shrieking* of the child constant and violent.

This, then, is the early progress of acute encephalitis. If the child have been healthy and plethoric, (and the study of diathesis is most important,) the form will usually be phlegmonous, and we may expect the products of acute inflammatory action. This form, the acute, is certainly often more amenable to very active treatment than that of less degree, occurring in those constitutions which, as we shall see, are predisposed to another form of termination; just as pneumonia in a plethoric habit may induce hepatization or abscess, while in a strumous system it will light up a latent tubercle.

If fatality occur at this stage, we discover one or more of these appearances:—Flakes of lymph or purulent matter deposited on, or diffused between, the membranes; close adhesions or adventitious tissues between the arachnoid and pia mater, or even between the dura mater and the cranial vault, its arachnoid lining being almost inseparable from it.

SECOND STAGE — SENSIBILITY IN DEFECT.

But there will now be set up, especially if not *essentially*, in peculiar diatheses, another train of symptoms.

The pupil, that had been almost a point, becomes dilated, and the eye-lid drops; the limbs become flabby, and hang listless; the breathing is very irregular and by fits; after a jerking inspiration there is often a deep sigh, and sometimes a slight snore. In older children, there is a reluctance and an inaptitude to articulate particular syllables, and the words are *clipt;* the child answers incoherently or in monosyllables, and shuns reply with a shake of the head; the burning heat diminishes; there is far less sensibility to touch; the pulse, obeying the general depression, becomes laborious, irregular, slow. The cry is now *moaning*, the most important and threatening language of complaint, being that of suffering, with debility. The pulse now becomes very feeble and uncountable; the child lapses into a state of coma or stupor, often attended with tetanic spasm and locked jaw. Soon after, relaxation of the sphincters occurs; we have profuse diarrhœa; effusion has supervened, and the child may soon die in convulsion. This course of the most *acute* form of hydrencephalus continues usually from twelve to twenty-five days.

When the tendency to disease is less decided, or the child placed in more favourable circumstances of atmosphere or nurture, the course of disease will often be more gradual or stealthy; indeed, so mingled often are the cerebral and abdominal symptoms, that the diagnosis may be most difficult, and the treatment consequently wavering and indecisive. The symptoms I have alluded to may one or more exist, but in a milder degree. The child does not seem to *enjoy itself*, but to be suffering under a state of general *malaise*, and if it be two or three years old, it is capricious and fretful, essaying often to amuse itself, and then directly desisting, and all this ere the anxiety of the parent be aroused. The note of wailing, however, the knitting of the brows, the aversion to light, the lopping of the head, and decided remissions of febrile disorder, lead to consultation, and the child is pronounced to be labouring under the first stage of the *insidious* form, as it has been termed, of encephalitis. To these signs succeed vomiting, constipation, parched lips, fauces, and nares, dry skin, and scanty and opaline urine. With all this, the pulse will be most fallacious, often for a time being quite regular, and then suddenly quick or flaccid. Then the eyes will be defective, one pupil being often contracted while the other is dilated.

These symptoms may continue thus in abeyance even for weeks under careful management, until some fresh excitement aggravates the disorder. It is from this protraction that the disease has been termed *chronic hydrocephalus*—improperly, for although the morbid action be protracted, yet its product, *effusion,* may prove very rapidly fatal. In early infancy, however, there is occasionally a remission or suspension of severe symptoms from another cause, the child becoming apparently relieved, even when the general signs are most unpropitious. The head will *enlarge* and become globular, the ingesta passing off crude and undigested, or in the form of small buttons in sour fluid, marasmus being progressive. This remission is the result of expansion of the cranial sutures, forming thus a safety valve to the brain, and averting the dangers of compression. I believe if recovery take place at this point, or rather, if effusion be arrested, especially if action continue, and, as is sometimes the case, pulmonary congestion be present, some anomalous change will often be induced. The cranial bones on the absorption of the fluid are not compact, and to compensate for this defect of space, an excess of nutrition takes place, and an hypertrophied and arid state of brain and membranes ensues, the meningeal vessels, after a time, being found almost bloodless. This is also the opinion of Otto, who usually found such condition in rickety children. This hypertrophy will sometimes progress from the first to the twentieth year. I must add, however, that I have seen one mark of hypertrophy, flattening of convolutions, combined with very extensive effusion. If the cranium ossifies during this excess of growth, we find the convolutions very firmly flattened against its vault. And when the calvarium is removed, the brain will overlap the skull. The prominent symptoms are convulsion, stertor, insensibility.

If we acknowledge the truth of these statements, do we not decide the question so often proposed, and so seldom satisfactorily answered—Is inflammation the cause of hydrocephalus? The question is puerile. As well may we inquire if pleuritis be the cause of hydrothorax. Effusion is doubtless the consequence of both, but not the *essential* consequence. If acute inflammation of the pleura occur in the plethoric, very active treatment will often rapidly subdue the action and ensure convalescence. Such, also, may be the result in the cerebral membranes; but in another diathesis, other effects will ensue. I have already implied my belief that in the great majority of encephalic effusions, the prevailing, if not the essential predisposing cause is strumous or tubercular tendency. Not that effusion is the invariable consequence of tubercular meningitis, for I have myself seen cases, and Dr. Bright records others, in which an *arid* condition rather of the membranes existed with tubercles and pulpy flakes. Nor have I reason to believe the opinion that tubercle is the coagulated solid of an albuminous pulp left after absorption of its watery particles. I have, however, so rarely witnessed fatal effusion without discovering some form or other of tubercle—often, indeed, combined with scrofulous sores, or cicatrices, or tumid glands, that I have been induced to believe it essential to that termination of the disorder we term acute hydrocephalus.

The true strumous child can scarcely be mistaken. The complexion is usually wan, pale, and dirty, the hair being either of a dull, light, tawny, or rusty black, and growing low on the forehead and the temples, while the capilluli are profuse over the whole body of the infant. The eyes are light grey or black, sometimes even *pinkish;* the muscles are flabby, and the heart acts feebly. This is the low form of struma, in which the depositions are either granular or caseous. The diathesis may exist, however, in a very different class—the fair, bright, and beautiful, with roseate cheeks, transparent skin, flaxen hair, and clear sparkling eyes, with highly developed front, and corresponding exaltation of intelligence, which makes them the admiration of parents and friends. But there is often latent poison in the system, and they must be watched with extreme jealousy if cerebral symptoms supervene. Even under the state of encephalic congestion, their extreme excitability will for a moment or two light up the mind; if left alone, especially, they often lapse quickly into coma. They are, indeed, usually characterized by extremes of slumber and wakefulness, or torpor and sensibility, these states alternating, as it were, in a direct ratio.

I have lately been attending, with my friend Mr. Smith, of Clapham-road, the daughter of Mr. L——, of Brixton, who was the subject of cerebral congestion, combined with subacute pneumonia. In her, these symptoms were very prominent; even while she was in much peril of effusion, she would start up in her bed and smile, and although her speech was affected, and she *clipped* her words, yet she engaged for a few moments in calm conversation; but no sooner were we out of her chamber, than she lapsed into comatose slumber, and we heard her snoring. This little girl was in sixty hours relieved of these oppressive symptoms, and so far out of peril.

The nature of the morbid deposition or development in these two forms of struma is not similar. In those very interesting children to which I have just alluded, it assumes the character of *fleshy* tubercle; it is usually encysted, and seated in or upon the *substance* of the brain, very frequently on the optic thalami; the symptoms to which it gives rise, if carefully watched, indicating the tissues on which it progressively encroaches.

Dr. Hooper has very ingeniously classified the isolated and encysted tumours of the brain, but in this essay it is useless to refine, as the symptoms depend more on the size and locality, than the peculiar nature of the morbid growth. I may state, however, that the three forms of tubercle which I have observed in children are the caseous, the fleshy, and the encephaloid, or medullary; the latter being rare and only in the broken-down or hyperæmiated brain. It is usually of a lobulated form, and its consistence unctuous.

The extent of these morbid growths and the consequent disorganization will often excite our wonder, convincing us of the truth of Dr. Baillie's assertion, that the severity of symptoms is often in an inverse ratio to the extent of disease, this, of course, depending on the degree of action, and, above all, the importance of the tissues involved. The truth is, that the encroachment is gradual, the vessels being by slow degrees compressed, and thus they with impunity compensate for pressure by the diminution of circulating fluid; for it must be remembered that the *volume* of the encephalon is not increased or diminished.

We see, therefore, in this form of complicated hydrocephalus a more protracted duration, a milder degree, and that which we might not anticipate, a frequent remission of symptoms, depending, I believe, on this balancing power of the circulation, and it may be from rapid absorption of fluid. Thus a child will be in a state even of stupor for a time, and then revive so far as to play with its toys, or seem to enjoy itself in painless tranquillity. This is, however, often but a flickering of the lamp of life, a calm between the storms, for the subjects of this fallacious state will often become suddenly comatose, and die. It is in these combined cases that we almost invariably witness paralysis, usually affecting the arm and leg of one side.

Miss M. L——, aged 7, the seventh child of the very healthy parents of an extremely fine and handsome family, was brought to me from Wandsworth. She had been for many months, and then was, under very excellent medical care, and had been occasionally seen by several physicians of the metropolis. She was now in a state of complete amaurosis, yet the pupils, as we sometimes see, were obedient to light. (The optic nerve may be compressed, although the lenticular ganglion be free.)

Her left thumb was pressed firmly to the outside of the index finger; there was almost constant opisthotonos in various degrees; there was a rolling in her gait when she could walk; at other times, and when she was lifted across the room, she dragged her leg after her.

With these very formidable symptoms, she was usually cheerful and chatty; her appetite was good, her digestion perfect, except when, from undue excitement, congestion ensued; then she became languid and silent, and averse to feeding. There were often, however, remissions of these symptoms. It was evident that there was pressure, solid or fluid, or both, about the optic thalami and pons Varolii.

The young lady lingered in this alternate state of depression and excitement, of suffering and apparent enjoyment, until winter, when she died, during a sudden attack of convulsion. We discovered a very extensive turgescence of the membranes, especially of the pia mater, its gorged vessels dipping down into all the convolutions, the vena Galeni and plexus being especially distended. On the optic track there was an hydatid of the size of a large pea, and on the corpora striata and optic thalami of the left ventricle, a large pink fleshy tumour of the size of a pigeon's egg, contained in a cyst, and pressing firmly on the optic nerve, which was much softened. On the pons Varolii there was a cyst containing half an ounce of fluid; the ventricles were distended by at least a pint of serum, covered merely by a thin fold of cerebrum; the septum lucidum could scarcely be recognised. The fluid was, however, nearly all in the right ventricle, yet dipping down into the infundibulum and into the third and fourth ventricles, in consequence of the tumour filling almost the left ventricular cavity. It was believed by the parents that the operation of a vascular nævus by ligature was the remote excitement of this tumour.

I may add that I have removed a very large mulberry nævus from the breast of the young lady's sister, by caustic, in consequence of the reluctance of the parents to the ligature.

The precocity of these little children should induce us to caution parents against over-stimulation of the brain, and to remind them that the child is too often made a plaything really for the amusement and delight of the mother or the nurse. It is certain that much mischief is thus induced, for if each admirer pets a child, the accumulation of excitement is extreme. So also premature culture of a precocious mind sooner or later lights up morbid vascular action; ghost stories also induce intense timidity and disorder in some. I have a little friend whose brain has been so stored, that he cannot walk alone in a street or a garden, without a sensation of extreme alarm.

As another illustration of Dr. Baillie's assertion, I may refer to the case of Mr. Griffith, in Dr. Bright's report. A portion of a tea-cup was driven into the brain, and there remained for two months, inducing abscess and hydrocephalus. The child did not seem to suffer acutely, but at length died in convulsion.

Regarding the essence of *tubercular* meningitis as the common predisposition to effusion, there will doubtless be difference of opinion. But I believe the presence of scrofulous deposition has been often overlooked. It is not always in flakes or large caseous masses; it is sometimes in the form of minute spheres embedded in the medullary tissue, but more frequently extensively scattered over the surface of the pia mater, but so diminutive, that unless the microscope be employed, or the finger very delicately drawn over the membranous surface, they may altogether elude discovery.

In December last I examined, with my esteemed colleague, Dr. Willshire, the head of a child fifteen months old. We were told it was healthy at the birth, but at the onset of dentition it began to be fretful, and was troubled with repeated attacks of diarrhœa, followed by rapid marasmus, mesenteric tabes. The head continued to enlarge, the common encephalitic and hydrocephalic symptoms were present and progressive until it died, with rigid opisthotonos. The fontanel, which had previously bulged, suddenly sunk as the child expired from reflux of blood from the sinuses to the heart. The mother had borne five children; three died when five months old, one at the fifteenth month, and one at the second year, with the same symptoms as the last. The mother's sister had borne sixteen children, and had only saved one of them, the fifteen having sunk under cerebral disease. The parents and many of the immediate relatives of these sisters were decidedly phthisical. Ossification of the cranium was proceeding at many points; there were several ossa triquetra. The dura mater adhered firmly to the calvarium; the brain appeared of healthy texture, *large and firm;* the convolutions flattened against the vault of the cranium. The ventricles, however, were perfectly unravelled and distended by limpid fluid, extending nearly the whole length of the hemispheres, about six inches. The convolutions were also partially unravelled towards the base. There were several small vesicles on the plexus choroides. The pia mater *appeared* healthy, but it was in reality studded by myriads of miliary granules, dipping down between the sulci (diffused tubercle), so small as almost to elude detection, except by careful and very delicate touch.

Those, however, who have perused the scientific pages of Dr. Hennis Green, especially in the *Lancet* of 1835, 1836, 1839, and 1840, will not now look for large fleshy deposits, or tubercular *masses*, ere they acknowledge the importance of this diathesis in cases of effusion.

There are other depraved conditions of the infantile fluids, which, I believe, may not only develop tubercle, but combining, perhaps, with the strumous blood, light up other morbid actions in the membranes, or induce ramollissement of the brain. I allude to that cachectic state which is induced by malaria, depraved diet, and defective or protracted lactation, a condition which so far justifies Cullen in placing cerebro-spinal effusions in the class cachexia, although he alludes merely to the chronic forms. The child thus poisoned will be seen to pine gradually, the egesta being crude and sour; an incessant diarrhœa of slime, often sanguineous, with small lumps indicating muco-enteritis; the gums spongy, the lips cracked, the skin livid; petechiæ often appearing and pervading even the cerebral membranes; ecchymosis being often found on the serous surface of the dura mater. This combination is, of course, a very hopeless condition.

### WASSERSCHLAG.

It is in these strumous conditions especially that the most sudden effusions sometimes take place; rarely without some warning, but with premonitory symptoms so slight as to be disregarded. We are told that a child has been running or playing about when it has been suddenly attacked by acute cephalitis, and has died in a few hours.

Usually, these effusions occur as the sequelæ of acute exanthemata, especially scarlatina. But it has seemed to me that the membranes of the brain will often take on a vicarious action on the recession of cutaneous eruptions, on the sudden check of diarrhœa, or the suppression of the renal secretion.

In these effusions, the water-stroke, or wasserschlag, there will often be no abnormal condition of the brain and its membranes; indeed, there may not have been time (although Goëlis thought differently) for the production of inflammation, the congestion, where it has occurred, having probably subsided, when much of the serum had been rapidly extravasated. In ischuria renalis, especially that combined with, or consequent on, the exanthemata, the incomplete desquamation of which suppresses diaphoresis, and of course oppresses the kidney, we often see rapid effusion into the cellular membrane, or in other cases between the cerebral tissues, even the dura mater and the skull. The rapidity both of effusion and fatality is striking, and the fatal result may perhaps be accelerated by the presence of urea in the blood of the brain; or, in cases of hepatic engorgement, by the portal blood, or the bile, if it be secreted, contaminating the circulating fluid.

The symptoms are accumulated by this rapidity. Thus, headach, dilated pupil, spasm of the globe, convulsion, opisthotonos, paresis, death—may all have supervened in the space of twelve or twenty hours. The fluid effused in these cases is usually watery serum, with not a trace of albumen. Although not so rapid in its onset or progress, effusion will also occur at the termination of malignant variola or acute infantile remittent, especially when the mucous membrane and glands are diseased; and also in adynamic fever, and in consequence of severe burns and scalds.

May we not, then, believe that the proximate cause of acute hydrocephalus is essentially encephalitis occurring in a system predisposed to effusion? I do not affirm that tubercular development is dependent on inflammation, but on the occurrence of this, active effusion is its result. One of my esteemed friends has, in his excellent essay, affirmed that there is no evidence of inflammatory action, but an effusion of clear serum; but is not this one of the essential terminations of inflammation, and it may so far unload the vessels as to leave the membranes free from any sign of increased vascularity.

But the morbid products of the very acute form will vary. The serum will often be highly coagulable, containing flakes of lymph, or floating pus, and sometimes layers of fibrine and albumen in the ventricles and sinuses. Goëlis asserts that he constantly saw, not only serum, but coagulable lymph, which covered the upper surface of the brain, and lined the walls of the ventricles. The seat of coagulated albumen is usually on the corpora striata and thalami, and in very young subjects, especially about the base of the brain, sometimes in the sella turcica, and even on the medulla oblongata. The pia mater is usually the seat of granular tubercle, especially about the folds of the choroid plexus; indeed, it is often separated by them from the medullary tissue, whereas in phlegmonous cephalitis in previously healthy children, the membranes of the cerebrum adhere more firmly than usual. The arachnoid is often turbid and opaline, especially about its reflexions, and in very severe cases it will become almost like white leather, adhering both to the dura and the pia mater.

The thalami and other tissues of the lateral ventricles will sometimes appear disorganized, the vessels seemingly shrunk away. The tubular neurine is sometimes studded with red points, and the hemispherical ganglion is darker than usual, (especially in precocious children,) sometimes, indeed, of a dull, modena-red hue; the outer surface of the skull, also, is often seen of a bright red tint.

The arachnoid cavity and subarachnoid tissue are the usual seats of effusion, the latter especially; the arachnoid often appears beautifully striated with vessels, which are, indeed, those of the pia mater, the nutrient membrane of the brain, from which the effusions are poured, and which also secretes the lymph that glues it to the arachnoid.

Sometimes the effusion does not extend beyond the veins of Galen downwards, being restrained by the reflexions of the membrane on those vessels. In the other case, the peculiar membrane of the ventricles secretes an excess of its natural halitus, extremely distending these cavities, and forming sacs, or hydatids, as they are mis-termed, on the plexuses.

The fluid, usually transparent, under the combination of hepatic engorgement, will become straw-coloured and of a darker yellow, tinging the encephalon even of a golden hue; in very acute cases it will even be found deeply sanguineous. This is often the case in the congestive inflammations consecutive to those disorders attended by impeded transmission of blood through the lungs, especially pertussis. Indeed, I believe that in twenty cases of fatal hooping-cough, at least fifteen die with cerebral symptoms, sometimes marked with hæmorrhage from the ear and nose. Dr. Bright relates a case where blood was effused on the brain from the exertion of cough.

These are certainly very formidable conditions, and I almost agree with Hufeland, that in these secondary cases meningitis is the herald of death, especially when the bronchial have for a time masked the cerebral symptoms.

Although I do not quite agree with Charpentier, or even with Dr. Abercrombie, in their exclusive opinions on this point, I believe it is in the protracted forms of congestion or subacute inflammation of the brain in the strumous diathesis, that we meet with those peculiar disorganizations we term ramollissement. That tubercle and softening are *relative* is highly probable, for we often see ramollissement in the phthisical adult. I believe not that mere cerebritis would induce this change, but rather hypertrophy or indurcissement: this, however, in the more energetic system.

The causes of softening appear to be either infiltration of the medulla (œdema of the brain) or an unravelling of the convolutions which I have seen extensively pervading the cerebellum; or, as in most cases, the detachment of the nutrient membrane from the medulla. We must remember that the brain of infants is naturally soft, and the slightest defect of nutrition is destructive. We have seen the surface of the brain where the membrane has been detached, a sort of yellow, creamy pulp, and this extending even to the degeneration of the corpus callosum. Regarding sanguineous effusion as a cause or consequence of softening, we must leave Rostan and his opponents to decide.

We have hitherto considered cerebral disorder as it is symptomatic, or the result of remote excitement. We will now allude to the morbid effects on the encephalon from contiguity or extension of disease, as otitis, and the accident of concussion.

I cannot write so decidedly regarding the extension of the disease from the nares, the æthmoid cells, or the frontal sinuses. I believe coryza maligna will often induce perilous and fatal disease within the cranium, the cerebral symptoms closely resembling the result of continuous otitis.

Sir Benjamin Brodie, I believe, affirms the commencement of the disease to be usually in the dura mater. It may be so occasionally, but I have so often proved the existence of otitis and otorrhœa before any cerebral symptom was present, that I cannot coincide in this decision.

The primary cause of this malady is usually fever, or scarlatina, or malignant sore throat, secondarily affecting the mucous passages. The result is ulceration of bone and cerebral abscess, or the deposition or growth of tubercle.

The occurrence of otitis is often for a time slighted, the attention being first drawn to a serous or muco-purulent discharge from the burst vesicles of otic herpes, or the pustules of impetigo. At first the pain may be slight, the concomitant symptoms mild; after a time the febrile state increases, the pain is more severe, and the discharge thicker and more purulent, yet still, perhaps, inodorous. As the disease progresses towards the tympanic membrane and the internal ear, pain and fever still increase, and at this point the spicula are often detached and dislodged, of which I have many cases.

When ulceration commences in the bone the pus is fœtid, and it will be fortunate if the course of the disease be towards the mastoid cells; but if the course be inwards the membranes are implicated, firm adhesion takes place between the dura mater and arachnoid, which is often of an oily condition; soon after, pus is formed, which lifts up the arachnoid from its adhesions, and the result is cerebral abscess. If erysipelatous or malignant sore throat be the primary diseases, the progress towards the encephalon will, if neglected, be more rapid.

I will briefly refer to a few cases of this description: a little girl, four years of age, was brought to me with puriform discharge from within and behind one of her ears, which had existed for many weeks, attended by pain, deafness, fever, startings, and moanings. The disease had not only progressed to the mastoid cells, but the internal ear was evidently deeply implicated. A ball of dead bone was detached from the mastoid process, and the symptoms became somewhat milder. Cascarilla, the mineral acids, the grey powder, and laxatives, were given, and poultices applied, but the child became delirious, and died in convulsion.

In this case, the internal ear, Eustachian tube, and mastoid cells, formed one cavity, and this communicated through the temporal bone by two carious holes, and with the cerebrum through the perforated membranes. In the left ventricle was a tubercle of the size of a walnut, which shelled out entire on slight pressure, and there was another of smaller size beneath the tentorium, embedded in the cerebellum.

Dr. R. Bennett related a case at the Medical Society, in which strabismus and slight convulsion were the only symptoms. A tubercle as large as a walnut involved almost the whole medulla oblongata—so far disproving one assertion, that there was greater severity of symptoms when disease was at the base of the brain.

My esteemed friend, Mr. Pilcher, exhibited at the same society, a short time ago, a portion of bone taken from a child in whom otitis had produced two cerebral abscesses, one in the left hemisphere, the other in a lateral ventricle; and related another case, in which the symptoms had so much abated as to afford confident hope, but this little girl died in her sleep. An encysted abscess was found in the cerebellum, containing four ounces of pus.

Herpetic ulceration spreading from the meatus externally, may, however, so destroy the bone as to be equally fatal. The scabrous patches of bone are usually on the squamous plate close to the petrous portion of the bone. I have a very fine specimen of this lesion before me, the particulars of which I do not remember. The abscess, however, was external, and detached the pericranium to the extent of a half-crown; there was a large perforation in the centre of the diseased spot.

In the strumous diathesis concussion will often light up action and develop the latent tendency. My friend Mr. Streeter has recorded the case of a girl, decidedly rickety, in whom a blow from a fall on the pavement was followed by ventricular effusion, the prominent symptoms being double vision and convulsion. This proved fatal in a month.

I was requested to see William Gems, aged seven, residing in Newington Butts, who had suffered, about three months before, a contusion on the side of his head. He had complained of occasional pain, was languid, and averse to amusement, and had lost his appetite. When I saw him there was convulsive action, occasional giddiness and vertigo, with slight paralysis. The bowels were not irregular. His cry was a scream, and occasionally a moan. On the bruised part of the cranium there was a painful and discoloured spot. He had constant remissions of fever, and about a week before I saw him he became convulsed, and died. The left parietal bone was of a dull brown hue for about the space of a half-crown, and there was a sort of coagulum between the laminæ—cephalhæmatoma. Beneath this, in the cerebrum, was imbedded a large globular tubercle, about one inch and a half in diameter, and in its centre there was a deep red nucleus. It was connected with the ceiling of the ventricle, but did not encroach on it.

We have thus alluded to the chief forms of cerebral disease during the very early stage of life. To accord with the conciseness of an essay we have not formally discussed the points of diagnosis and etiology, but would refer to the symptoms by which each form is characterized.

A word, however, on the forms and stages of hydrencephalus, on which Cheyne, Whytt, Goëlis, Itard, and others, have commented so profusely. Copland asserts the arrangement of Cheyne, which is indeed a symptomatological division into three:—increased and diminished sensibility and convulsion; the proximate causes of which, irritation, inflammation, effusion, and compression, so far coinciding with those of Goëlis—turgescence, inflammation, effusion, and palsy. Irritation may, however, exist for a time without congestion, therefore the addition and premonitory stage of irritation, which really constitutes the *nervous* form of the disorder, will render the blending of these arrangements perfect.

Still there is much error in our decision in the early stages; it is in this that pathologists have so often been deceived. The difficulty is fairly confessed by one of the most scientific symptomatologists, in his excellent Dictionary. The irritation of ascarides may, for instance, assume every symptom of the stage of irritation, intestinal disease, congestion, &c. &c. I therefore waive the discussion of a formal diagnosis.

In every child, but especially those predisposed to cerebral disease, the subject of PROPHYLAXIS is most important. Judicious nurture will control or subdue; the contrary mode will favour or even induce, the development of predisposition. Both in prophylaxis and in cure, however, it is ever essential to study the constitution of a child, as the same precept will not always be appropriate. Air and exercise, clothing, diet, and modes of nursing, however, are points of importance in every case, but especially in those of tubercular diathesis, which form so large a portion of them. To ensure for all exercise in warm, dry days, to adopt a regular and moderate mode of feeding, to keep the head cool and the feet warm, and to regulate the functions of the alimentary canal, are points which should never be disregarded, especially during the perilous period of dentition. *Repletion,* especially, will obstruct the motion of the diaphragm and excite vomiting, and the dry *stomach*

*cough;* it may compress the bile ducts, produce congestion of the head, and thus directly become a cause of disorder.

When the evacuations are in any way disordered, the grey powder, with rhubarb and chalk, according to their consistence, frequently may be safely administered; and if the child be weakly, or of the strumous diathesis especially, combined with the syrup of the iodide of iron. If there be tenderness of the abdomen, fomentations or warm sponges may at any time be employed.

The child should be almost constantly in a state of repose, and not, for the sake of amusement, be suddenly excited. If it *wishes* to slumber, it should never be kept awake. Its head should not lie low; and in nursing or playing with it, it should not be tossed or whirled about, or patted violently, as is often done, on the back.

We should be very cautious, also, during teething, with regard to external remedies for cutaneous disease. My learned colleague, Dr. Copland, and myself have seen repeated instances of the peril in which a child is placed by such treatment. These eruptions are often established on the skin, as a safety valve—a truth conversely proved by the relief so often afforded by their reproduction, as a form of counter-action, as well as by a seton or issue.

The TREATMENT of encephalitis will of course depend on its causes, its stages, and on constitution or diathesis. I will, in allusion to it, adopt the form of propositions rather than a more elaborate discussion.

In the acute form of phlegmonous inflammation of the brain, or its membranes, and in the first stage the remedies should ever be decidedly antiphlogistic. In the plethoric child of two years old, the jugular vein may be opened. Above this age it will, for a delicate hand, be as easy and more safe to bleed from the arm, or leeches may be applied *behind* the ear, or a small cupping-glass to the forehead. I recommend the application of leeches behind the pinna of the ear, as pressure there may easily check hæmorrhage, and it is the closest available point to the exit of the internal jugular from the foramen. It would be presuming to decide abstractedly regarding the quantity of blood to be drawn; the circumstances of the case can alone decide. If, however, the pupil be a mere point, or much contracted; the skin hot and dry; the pulse quick and full; the fontanel elevated, we should not certainly be too lenient. The *ounces* may usually be fairly measured *by the years* in robust children, or we may be very well guided by pallor, languor, and approach to syncope.

In my own practice, I always regard the fontanel when contemplating depletion, and I may glance at two recent cases in illustration. The one was in a child of Mr. S——, of Newington, labouring under all the prominent symptoms of acute arachnitis; the fontanel was in the *form of a ball.* I did not hesitate to take blood until the child became languid and pale. In the other, a child of Mr. B——, of Clapham-road, whom I saw in consultation, the same *general symptoms* were present, but the *fontanel was depressed,* and the child was of less robust constitution. I did not bleed here. Both the children soon recovered. I believe a different plan with each would have been followed by a different result.

If the gums are *spreading,* while the mouth is hot and dry, free incision should be effected, and the blood absorbed by a small soft sponge, so that it be not swallowed. The feet should be enveloped in warm socks, and ether water applied constantly on folded rag along the sagittal suture. If the symptoms continue, the more refrigerant effect of ice in a bladder or a caoutchouc cushion may be resorted to; or a stream of cold water may be poured on the crown. We may sometimes witness the dilatation of the pupil as this effect proceeds. The refrigerant plan must yet be restricted to time, as asphyxia has often occurred from its undue employment. If the bowels be confined, an active aperient should be given, and after a few hours a warm enema of salt and water.

These are the *immediate* remedies to be adopted if we are summoned early to the case. A judicious modification, however, must be made if the symptoms be of the subacute form, or have longer existed, or if the disorder be consecutive of acute disease, in which debility has been induced.

In those forms which have been called nervous, depletion must be very guarded; the formation of vesication by the acetum lyttæ in three or four points behind each ear, and on the nuchæ, will often be preferable, especially as we can then, without irritating the bowels, affect the system with mercury by the endermic method.

In some cases we may prefer a more powerful rubefacient: castor oil, with liquor ammoniæ, or friction with camphor and ammonia liniment with mercurial ointment, especially inside the legs. If convulsion or fit at any time supervene, a few drops of tincture of assafœtida in warm water may be given twice or thrice in the hour. It is especially useful, almost essential, in the low nervous form.

The next indication is to regulate the secretions of the liver, the skin, and the kidneys. If there be no contraindication, from one to four grains of calomel may be given every two, three, or four hours, according to the age, until a scruple is introduced, (not, however, as a purgative, as Dr. Abercrombie seems to have employed it.) The excess of mucus renders the young child capable of taking large doses of this mineral. It must be confessed, however, that occasionally much mucous irritation is induced by it.

The combination of liquor ammoniæ acetatis, spiritus etheris nitrici, and antimonium tartarizatum, will usually regulate the other secretions.

It will often be requisite to combine the calomel with other remedies. Thus, if diarrhœa be present, chalk or Dover's powder may be added.

If there be debility or exhaustion, or if the habit be cachectic, or if prostrate coma supervene, camphor may be combined, from a quarter to half a grain, twice or thrice in a day.

If vomiting continue after the urgent symptoms are relieved, magnesia, with lemon juice, or fluid magnesia, with half a drop or more of Battley's sedative solution, may often afford relief. It is in the *protracted* case especially, where free depletion is contraindicated, that stimulating enemata are so valuable. A drachm of castor oil, and half a drachm of oil of turpentine, may be given in thin gruel, even to the infant, often, with the effect of inducing quietude, or the removal of a comatose condition.

In those cases consecutive to scarlatina or other exanthemata depletion, though usually essential, must be more limited. The quantity should be very small, and repeated two or three times at intervals. It is in these cases that digitalis is so valuable a remedy, especially if combined with very small doses of calomel. As there is often, however, much debility, the syrup of iodide of iron may be combined with great benefit twice or thrice in a day, and this without the imputation of a paradox.

When otitis occurs in children, we should ever be watchful of the ultimate results. Leeches should be freely applied on the mastoid process, and after twenty-four hours vesication induced on the same spot, the sore being kept open with savine or mercurial ointment. In some cases of great prostration, dry cupping may be more advisable. The other antiphlogistic remedies I need not refer to.

If suppuration should be suspected, linseed poultice, or the newly-adopted *piline,* should be assiduously applied, with the aim of soliciting the course of pus towards the mastoid cells, a comparatively unimportant spot; and if abscess points there, or we can detect pus, an opening should be made early, or deep incision on the cells, or the trephine may be used, if we can safely reach the abscess by such a mode: it is of vital importance to prevent the *inward* progress of matter.

When encephalitis has continued for several days unrelieved, and has not, by its interference with the functions of the brain, or by exhaustion, been fatal, the second stage or result of the disorder will have supervened. This *maybe* suppuration is rare in very young children; the more common result is effusion of serum.

Even after this deposition, acute symptoms may still call for active treatment, especially the continuation of decided counteraction, by sinapism or stimulant liniments, especially inside the legs, and mercurial inunction. Although we confess that the prognosis must now be most unfavourable, it is our duty to *persevere*. *Repetition* of bleeding is usually, not always, injudicious.

When action is subsiding, the iodide of potassium will be most beneficial in doses of from one grain to four or six grains, thrice in a day. Sarsaparilla also will be often beneficial, or the infusion of serpentary—a very valuable remedy.

In emaciated children, the syrup of iodide of iron, or citrate of iron in infusion of orange-peel and cascarilla, may be resorted to; and all this may be combined with iodine embrocation on the head.

It is in the protracted or chronic stage that puncture of the membranes may be resorted to. In Dr. Conquest's operations, I believe about one-fourth part survived. I confess I have not proved it so favourable. Nor can I write decidedly on the subject of compression, or the bandages of Trousseau.

The diet should consist of asses' milk; for older children, the mucilages and jellies, with grapes, currants, and other subacid fruits. The most nutritious diet, I believe, consists of chicken-jelly, combined with arrow-root or isinglass, or Previte's Ceylon moss.

After complete convalescence is attained, there may still be excessive irritability. In these cases, we shall find benefit from quinine, combined with Dover's powder, in small doses, with a drop or two of muriatic acid in infusion of cascarilla, for older children, thrice in a day. It cannot be doubted that fresh air, especially that near the sea, will be highly conducive to the confirmation of health.

Regarding the treatment of hypertrophy, atrophy, indurcissement, and softening, I consider them so much in the light of irremediable or established conditions, that I scarcely think them obedient to remedy. Dr. Sims has written very confidently on the cure of ramollissement. His evidences of recovery are, pin-hole or honeycomb texture of brain, or small serous cysts.

In those cases of paresis in which the cerebro-spinal axis has been much implicated, we may often be for some time disappointed in our treatment; but there is much difference as to power of recovery in different nerves. The paresis of the fibrillæ of the facial, for instance, producing distortion on one side, often recovers quickly, while that of the limbs is protracted. Of the former, I have now a very interesting case, which is nearly cured within eight months, although at one time the excessive distortion of one side, in consequence of the want of muscular antagonism on the other, was really frightful.

I was called, two years ago, to the infant of a merchant in the City, who was suddenly attacked by convulsion during dentition, almost without warning; yet the fits *recurred* frequently. Warm bath, calomel, assafœtida, ether lotion, and free incision of the gums, soon restored her to a safe condition, and I sent her into a purer air in the country. She is now returned a very fine, healthy, rosy girl, most animated, good-tempered, and *loving*, yet with some suspicion of slight fatuity. Her speech is defective; indeed, she cannot pronounce a word properly; the left arm and leg are cold, flabby, and almost powerless; her grasp is very feeble, and the most powerful stimulating liniments &c. have not been yet successful.

In the most severe forms of paresis, galvanism will sometimes be a very valuable agent. On the principle of excito-motory influence in inducing more healthy innervation, frequent tickling of the sole or the palm may be employed; for it is certain that not only may a paralyzed muscle be thus excited to *involuntary* action, but that, in consequence of increased vascular action, a limb, to a certain degree atrophied, may be restored to its healthy condition. Medicine, which in the adult is often profitable, is of little use in young children, and we must often be content to send them to the sea-side, into a purer air, and trust to that influence and time for their recovery.

